# Postpartum depression and risk factors among working women one year after delivery in Beijing, China: a cross-sectional study

**DOI:** 10.3389/fpubh.2024.1346583

**Published:** 2024-05-02

**Authors:** Jian Zhao, Min Zhang

**Affiliations:** School of Population Medicine and Public Health, Chinese Academy of Medical Sciences and Peking Union Medical College, Beijing, China

**Keywords:** working women, postpartum depression, risk factors, commercial enterprises, maternal mental health

## Abstract

**Background:**

Postpartum depression (PPD) is the most common mental illness affecting women after childbirth, and working mothers may be faced with unique challenges. This study aimed to examine the depression status among working postpartum women in 1 year of childbirth and explore the relationship between occupational factors and PPD in urban Beijing, China.

**Methods:**

A cross-sectional survey of 554 postpartum women was conducted among ten community health service centers in six urban districts of Beijing, China. Sociodemographic, occupational, childbirth and postpartum information were collected. Depression status was obtained using the Edinburgh Postnatal Depression Scale. Prevalence of postpartum depression (PPD) was assessed in relation to occupational characteristics, and influencing factors were analyzed through logistic regression.

**Results:**

Of the postpartum women, 29.42% met the criteria for depression. PPD prevalence was significantly higher among women employed in commercial enterprises (39.81%). The analysis of influencing factors showed that age, family or personal monthly income, maternity leave, feeding methods, and postpartum care affected the psychological health of occupational women after childbirth.

**Conclusion:**

PPD prevalence is notably elevated among women employed in commercial enterprises, and specific risk factors contribute to its occurrence. These findings highlight the need for targeted interventions to address these risk factors and prevent PPD in this population.

## 1 Introduction

China has the largest number of female workers in the world, representing approximately 26% of the global female employment total (1). The female employment rate in China also ranks among the highest worldwide. According to data from the National Bureau of Statistics of China, in 2021, there were 320 million employed women in the country, constituting 43.1% of the total workforce (2). This proportion consistently surpasses that of Asia-Pacific nations such as South Korea (42.7%) and Singapore (41.2%), as well as the global average (39.3%). It is comparably aligned with that of Western developed countries such as the United States (46.2%) and Sweden (47.4%) (1). The health of these female workers, particularly in terms of mental well-being, is a critical concern.

Postpartum depression (PPD) is the most common mental health issue affecting women after childbirth, with symptoms persisting for up to 1 or 2 years (3). PPD symptoms, such as sadness, irritability, anxiety, and poor appetite, can escalate to severe agitation and in extreme cases, self-harm or infanticide (3, 4). This condition not only affects the well-being of the mother but also poses significant risks to the newborn and affecting the socio-emotional dynamics of the family. This underscores the necessity for proactive attention and effective interventions.

Well-established risk factors associated with developing PPD include age, low education level, low socioeconomic status, limited social support, relationship difficulties, a history of depression and depression during the pregnancy (5, 6). Hormonal fluctuations, nutritional deficiencies, sleep disturbances are also contributing factors to PPD (7). While these risks are universal among all new mothers, working mothers are faced with unique challenges. Typically, most professional women return to work within the first year post-delivery, which largely depended on the duration and terms of maternity leave. The features of maternity leave, such as length, wage replacement and employment protection, significantly impact maternal mental health (8, 9). Moreover, workplace conditions, including work schedules, shift work, reward structures, and work–family conflict, can act as stressors, heightening the risk of depression (10). Due to the dual demands of infant care and employment, working mothers are particularly susceptible to workplace stress.

Maintaining good psychological and emotional health is essential for job performance and minimizing absenteeism. Studies reveal that PPD is associated with increased workplace absenteeism, reduced job performance, and higher disability costs for employers (11). Mothers who experience PPD often face heightened absenteeism, prolonged medical disability following childbirth, difficulties in re-adjusting to the work, and heightened relationship conflicts, including divorce (12).

Despite the importance of this issue, current research has insufficiently addressed the experiences of female workers, especially in China. While some studies have explored PPD among working mothers (13–15), they have not considered the specific characteristics of occupations and how these might impact PPD. Given the profound implications of PPD on maternal health, child development, and workplace dynamics, this study aims to analyze the prevalence of postpartum depression among women in different occupational categories in urban Beijing within 1 year of childbirth, and to explore the relationship between occupational factors and PPD. The research seeks to provide a comprehensive understanding of the factors influencing PPD in the context of working mothers, thereby guiding the development of targeted strategies to mitigate risks and support recovery.

## 2 Materials and methods

### 2.1 Study setting and design

This research was conducted in six urban districts of Beijing, the capital city of China. Postpartum women are required to establish maternal and child health records within 1 week of childbirth at community health service centers. Over the following 3 years, these centers provide health checkups for infants in every 6 months, and vaccination services. Data were collected when a mother accompanied her one-year-old baby to the health center for a physical examination or vaccination. If the mother was not present, we contacted them by phone and sent the survey questionnaire via WeChat or SMS to complete the data collection.

### 2.2 Participants and recruitment

Cluster sampling was used to conduct a cross-sectional survey. Inclusion criteria of Postpartum women: (a) those who established maternal and child health records from November 2017 to June 2018 in 10 community health service centers across the six urban districts of Beijing; (b) women who were in the 1 year postpartum, with normal communication and understanding abilities; (c) those who voluntarily participated in the survey and signed an informed consent form. Exclusion criteria: Those previously or during pregnancy diagnosed with organic brain disease; those with a history of psychiatric disorders; and those who refused to participate after explanation. This study was approved by the Ethics Review Committee of the Institute of Basic Medical Sciences, Chinese Academy of Medical Sciences (067-2021).

### 2.3 Data collection

Data on these women for 1 year postpartum were collected from November 2018 to June 2019. After the respondents signed the informed consent form, they were enrolled in the study. Data collection was initiated with a digital questionnaire. A total of 589 cases met the inclusion criteria for the study, with 554 cases ultimately providing valid data, with an effective response rate of 94.06%.

### 2.4 Measurements

One-year postpartum data were collected using a two-part questionnaire. Part 1 focused on sociodemographic profile (age, education level, family and personal monthly income), employment factors (the women’s occupational status and maternity leave), and postpartum factors (baby’s health status, baby’s feeding methods, and the women’s caregiving status). Perinatal information such as mode of delivery, parity, and complications were recorded from the maternal and child health records in the community health center.

Part 2 was the Edinburgh Postnatal Depression Scale (EPDS) for mental health status measurement. The Chinese version of EPDS was used to determine the occurrence of PPD. The EPDS has a sensitivity of 85%, specificity of 77%, positive predictive value of 83%, split-half reliability of 85%, and a Cronbach’s α coefficient of 0.87 (16). The subjects used a self-assessment method, selecting corresponding scores (0-never, 1-sometimes, 2-often, 3-always) on 10 items (mood, pleasure, self-blame, anxiety, fear, insomnia, coping ability, sadness, crying, and self-harm) (17). A total score of ≥13 points was diagnostic for PPD.

### 2.5 Classification of postpartum women’s occupations

Referring to the occupational classification in socio-economic status studies (18–21), the occupational information collected from questionnaire was categorized into four groups: physical workers (PW, workers, agricultural/forestry/fishery/water conservancy production personnel, cleaning and sanitation workers, restaurant service personnel, retail salespersons, etc.), commercial enterprises (CE, state-owned enterprises, foreign-funded enterprises, and small and medium-sized enterprise workers, financial insurance and other commercial practitioners, etc.), professionals, government office & public institution employees (PGPE, medical staff, teachers, scientific and technical workers, cultural workers, professional and technical personnel, staff of state organs and institutions, etc.), and unemployed or self-employed (US).

### 2.6 Data analysis

All data were checked before entry and were double-entered, with statistical analysis performed using SPSS software version 27.0. Count data were described using frequency (percentage), analyzed using the χ^2^ test, and post-hoc tests were conducted using the Bonferroni method for pairwise comparisons between groups; logistic regression analysis with forward stepwise was used to analyze factors related to PPD. The level of significance was α = 0.05, with *p* < 0.05 considered statistically significant.

## 3 Results

### 3.1 General characteristics of postpartum women in four occupational categories

Among 554 postpartum women surveyed within 1 year after delivery, the majority were aged 30–34 years (35.56%). Among these, the unemployed or self-employment under the age of 25 were more prevalent (38.61%), while women working in commercial enterprises above the age of 35 were more common than in other groups (23.79%). The majority had received tertiary education, either at a college or undergraduate level (83.94%), with physical laborers and the unemployed or self-employed women more likely to have a high school education or less, compared to other occupations. The predominant range for family monthly income was between 10,000 and 30,000 RMB (54.69%), with women in commercial enterprises reporting higher family incomes than other categories (53.88%). Personal monthly income most commonly fell between 5,000 and 15,000 RMB (78.52%), with those in the commercial sector earning more than their counterparts in other categories. Additionally, women employed in commercial enterprises had a higher proportion of first childbirths, pregnancies that were well-prepared, mixed feeding practices, and hiring of nannies compared to other occupational categories. See Table 1 for details.

**Table 1:** Characteristics and univariate analysis among four occupational categories of postpartum women.

Characteristics	All		PW		CE		PGPE		US		χ^2^	*p*
	(*N*, %)		(*N*, %)		(*N*, %)		(*N*, %)		(*N*, %)			
Age (year)											102.356	<0.001
<25	61	11.01	4	5.41	12	5.83	6	3.47	39	38.61		
25–29	191	34.48	29	39.19	73	35.44	70	40.46	19	18.81		
30–34	197	35.56	30	40.54	72	34.95	66	38.15	29	28.71		
>35	105	18.95	11	14.86	49	23.79	31	17.92	14	13.86		
Education level											61.603	<0.001
Senior school or less	37	6.68	15	20.27	7	3.4	2	1.16	13	12.87		
College or university	465	83.94	57	77.03	181	87.86	142	82.08	85	84.16		
Graduate	52	9.39	2	2.70	18	8.74	29	16.76	3	2.97		
Monthly family income (RMB)											107.630	<0.001
<10,000	89	16.06	20	27.03	20	9.71	32	18.50	17	16.83		
10,000–30,000	303	54.69	44	59.46	75	36.41	106	61.27	78	77.23		
>30,000	162	29.24	10	13.51	111	53.88	35	20.23	6	5.94		
Monthly personal income (RMB)											19.799	<0.001
<5,000	85	15.34	17	22.97	30	14.56	15	8.67	23	22.77		
5,000–15,000	435	78.52	54	72.97	157	76.21	148	85.55	76	75.25		
>15,000	34	6.14	3	4.05	19	9.22	10	5.78	2	1.98		
Parity											18.539	0.005
1	436	78.70	57	77.03	173	83.98	135	78.03	71	70.30		
≥2	118	21.30	17	22.97	33	16.02	38	21.97	30	29.70		
Pregnancy preparation											58.627	<0.001
Unintended pregnancy	68	12.27	14	18.92	11	5.34	26	15.03	17	16.83		
Planning for pregnancy but inadequate	215	38.81	20	27.03	68	33.01	63	36.42	64	63.37		
Fully preparation for pregnancy	271	48.92	40	54.05	127	61.65	84	48.55	20	19.80		
Mode of delivery											8.264	0.219
Natural labor	383	69.13	65	87.84	168	81.55	145	83.82	91	90.1		
Cesarean section or Vaginal assistant delivery	171	30.87	9	12.16	38	18.45	28	16.18	10	9.9		
Baby’s health condition											6.664	0.353
Good	469	84.66	65	87.84	168	81.55	145	83.82	91	90.10		
Some problem in health	84	15.16	9	12.16	38	18.45	27	15.61	10	9.90		
Feeding method											36.839	<0.001
Exclusive breastfeeding	240	43.32	44	59.46	73	35.44	92	53.18	31	30.69		
Formula or mixed feeding	314	56.68	30	40.54	133	64.56	81	46.82	70	69.31		
Maternity leave											8.574	0.199
4 months or less	177	31.95	22	29.73	65	31.55	61	35.26	29	28.71		
4–6 months	313	56.50	39	52.70	118	57.28	90	52.02	66	65.35		
6 months or more	64	11.55	13	17.57	23	11.17	22	12.72	6	5.94		
Postpartum care											44.336	<0.001
Hiring a nanny	76	13.72	0	0.00	49	23.79	20	11.56	7	6.93		
Assistance from relatives	341	61.55	48	64.86	101	49.03	120	69.36	72	71.29		
Only the couple	137	24.73	26	35.14	56	27.18	33	19.08	22	21.78		

PW, physical workers; CE, commercial enterprises; PGPE, professionals, government office & public institution employees; US, unemployed or self-employed.

### 3.2 Prevalence of PPD among postpartum women of four occupational categories

Based on an Edinburgh Postnatal Depression Scale (EPDS) score of ≥13, the overall prevalence of PPD among the surveyed subjects was 29.42% (163/554 individuals). The prevalence of PPD in women employed in commercial enterprises was 39.81% (82/206 individuals), which was significantly higher than that of other occupational categories, with a statistically significant difference (χ^2^ = 19.284, *p* < 0.01). See Table 2 for details.

**Table 2:** The prevalence of PPD among four occupational categories of postpartum women.

Occupational groups	All (*N*, %)		PPD (*N*, %)		Non-PPD (*N*, %)		χ^2^	*p*
							19.284	<0.001
PW	74	13.36	14	18.92	60	81.08		
CE	206	37.18	82	39.81	124	60.19		
PGPE	173	31.23	38	21.97	135	78.03		
US	101	18.23	29	28.71	72	71.29		
All	554		163	29.42	391	70.58		

PW, physical workers; CE, commercial enterprises; PGPE, professionals, government office & public institution employees; US, unemployed or self-employed.

### 3.3 Univariate analysis of PPD among postpartum women

Univariate analysis revealed that women over the age of 35 have a significantly higher proportion of postpartum depression (PPD) at 41.90% compared to other age groups (χ^2^ = 11.860, *p* = 0.008). Families with an income between 10,000 to 30,000 RMB show a lower prevalence of PPD at 24.09% (χ^2^ = 9.860, *p* = 0.007). Women with unintended pregnancies show the highest rate of PPD at 47.06%, which is statistically significant (χ^2^ = 14.899, *p* = 0.001). Mothers of babies with health problems have a significantly higher rate of PPD compared to mothers of healthy babies (χ^2^ = 20.567, *p* < 0.001). Women who choose formula or mixed feeding have a higher proportion of PPD at 35.67% (χ^2^ = 13.632, *p* = 0.001). The prevalence of PPD among mothers who hire a nanny is significantly higher at 49.15%, compared to those who receive postpartum care solely from the couple themselves or with assistance from relatives (χ^2^ = 25.873, *p* < 0.001). See Table 3 for details.

**Table 3:** Univariate analysis of PPD among postpartum women.

Characteristics	PPD		No-PPD		χ^2^	*p*
	(*N*, %)		(*N*, %)			
Age (year)					11.860	0.008
<25	12	19.67	49	80.33		
25–29	56	29.32	135	70.68		
30–34	51	25.89	146	74.11		
>35	44	41.90	61	58.10		
Education level					5.297	0.071
Senior school or less	2	18.18	9	81.82		
College or university	28	22.05	99	77.95		
Graduate	133	31.97	283	68.03		
Monthly family income (RMB)					9.860	0.007
<10,000	29	32.58	60	67.42		
10,000–30,000	73	24.09	230	75.91		
>30,000	61	37.65	101	62.35		
Monthly personal income (RMB)					0.245	0.885
<5,000	24	28.24	61	71.76		
5,000–15,000	130	29.89	305	70.11		
>15,000	9	26.47	25	73.53		
Parity					2.050	0.153
1	122	27.98	314	72.02		
≥2	41	34.75	77	65.25		
Pregnancy preparation					14.899	0.001
Unintended pregnancy	32	47.06	36	52.94		
Planning for pregnancy but inadequate	67	31.16	148	68.84		
Fully preparation for pregnancy	64	23.62	207	76.38		
Mode of delivery					0.618	0.734
Natural labor	111	28.98	272	71.02		
Cesarean section or Vaginal assistant delivery	53	30.99	119	69.59		
Baby’s health condition					20.567	<0.001
Good	121	25.80	348	74.20		
Some problem in health	41	48.81	43	51.19		
Feeding method					13.632	0.001
Exclusive breastfeeding	51	21.25	189	78.75		
Formula or mixed feeding	112	35.67	202	64.33		
Maternity leave					1.416	0.702
4 months or less	32	27.35	85	72.65		
4–6 months	95	30.35	218	69.65		
6 months or more	16	25.00	48	75.00		
Postpartum care					25.873	<0.001
Hiring a nanny	29	49.15	30	50.85		
Assistance from relatives	100	23.87	319	76.13		
Only the couple	34	44.74	42	55.26		

### 3.4 Logistic regression analysis of occupational-related influencing factors of PPD in postpartum women

In the regression analysis, occupation was initially incorporated as an independent variable to explore whether any specific occupational group was associated with an increased risk of PPD in the sample. After adjusting for age, education level, and monthly family income, and further adjusting for pregnancy preparation, baby’s health condition, breastfeeding method, and postpartum care, it was found that employees in commercial enterprises (OR = 4.184, 95% CI: 1.036–5.247, *p* = 0.041) were positively associated with an increased risk of PPD compared to the US group (Table 4).

**Table 4:** Logistic regression analysis of occupational groups affecting PPD among postpartum women.

				Model 1^a^			Model 2^b^		
	OR	95% CI	*p*	OR	95% CI	*p*	OR	95% CI	*p*
US	1			1			1		
PW	0.998	0.422–2.365	0.997	0.704	0.322–1.537	0.378	0.921	0.365–2.325	0.862
CE	2.126	1.124–4.022	0.020	1.952	1.060–3.595	0.032	2.105	1.021–4.339	0.044
PGPE	1.271	0.646–2.501	0.488	0.700	0.371–1.322	0.272	1.036	0.488–2.197	0.927

a Model 1: Adjusted for age, education level and monthly family income.

b Model 2: Further adjusted for pregnancy preparation, baby’s health condition, feeding method and postpartum care.

PW, physical workers; CE, commercial enterprises; PGPE, professionals, government office & public institution employees; US, unemployed or self-employed.

Using PPD as the dependent variable, further logistic regression analysis was conducted with family monthly income, personal monthly income, length of maternity leave, care condition, and feeding method as independent variables. The results, after adjusting for age, educational level, and further adjusting for perinatal factors such as parity, whether the pregnancy was well-prepared, and baby’s health status, indicated that factors significantly associated with PPD are presented in Table 5.

**Table 5:** Logistic regression analysis of variables affecting PPD in four occupational groups of postpartum women.

Variables				Model 1^a^			Model 2^b^		
	OR	95% CI	*p*	OR	95% CI	*p*	OR	95% CI	*p*
PW									
Monthly family income (yuan)									
10,000–30,000 vs. <10,000	7.446	0.038–0.586	0.006	7.615	0.034–0.564	0.006	4.658	0.035–0.851	0.031
CE									
Monthly personal income (yuan)									
5,000–15,000 vs. <5,000	4.808	1.572–14.706	0.006	4.250	1.374–13.142	0.012	3.326	1.021–10.839	0.046
Maternity leave									
4 months or less vs. 6 months or more	2.189	0.568–8.437	0.255	2.395	0.610–9.405	0.211	4.851	1.118–21.059	0.035
4–6 months vs. 6 months or more	5.008	1.361–18.424	0.015	5.839	1.549–22.005	0.009	9.852	2.352–41.263	0.002
Postpartum care									
Assistance from relatives vs. Only the couple	5.248	0.231–0.892	0.022	5.754	0.202–0.851	0.016	4.426	0.209–0.946	0.035
Feeding method									
Formula or mixed feeding vs. Exclusive breastfeeding	2.479	1.210–5.079	0.013	2.322	1.118–4.821	0.024	2.411	1.110–5.239	0.026
PGPE									
Feeding method									
Formula or mixed feeding vs. Exclusive breastfeeding	6.481	1.260–5.909	0.011	6.082	1.225–5.908	0.014	4.184	1.036–5.247	0.041
Care of maternal and infant									
Assistance from relatives vs. Only the couple	4.188	0.179–0.964	0.041	3.989	0.174–0.984	0.046	5.506	0.135–0.836	0.019
US									
Feeding method									
Formula or mixed feeding vs. Exclusive breastfeeding	11.489	0.073–0.497	0.001	3.707	0.124–1.019	0.054	4.014	0.111–0.976	0.045

a Model 1: Adjusted for age and education level.

b Model 2: Further adjusted for parity, pregnancy preparation and baby’s health condition.

PW, physical workers; CE, commercial enterprises; PGPE, professionals, government office & public institution employees; US, unemployed or self-employed.

For physical workers, a family monthly income between 10,000 and 30,000 RMB was negatively correlated with the occurrence of PPD (OR = 0.035–0.851, 95%CI: 4.658, *p* = 0.031). For women employed in commercial enterprises, personal monthly incomes between 5,000 and 15,000 RMB (OR = 3.326, 95%CI: 1.021–10.839, *p* = 0.046), maternity leave of less than 4 months (OR = 4.851, 95%CI: 1.118–21.059, *p* = 0.035), maternity leave between 4 and 6 months (OR = 9.852, 95%CI: 2.352–41.263, *p* = 0.002), and mixed or formula feeding (OR = 2.411, 95%CI: 1.110–5.239, *p* = 0.026) were positively correlated with the occurrence of PPD; whereas having relatives to help with care (OR = 0.209–0.946, 95%CI: 4.426, *p* = 0.035) was negatively correlated with the occurrence of PPD. For women in professionals, government office & public institution, mixed or formula feeding (OR = 4.184, 95%CI: 1.036–5.247, *p* = 0.041) was positively related with PPD; and having relatives to help with care (OR = 0.135–0.836, 95%CI: 5.506, *p* = 0.019) was negatively correlated with the occurrence of PPD. For the unemployed and self-employed, mixed feeding (OR = 0.111–0.976, 95%CI: 4.014, *p* = 0.045) was negatively correlated with the occurrence of PPD.

## 4 Discussions

### 4.1 High prevalence of PPD in urban Beijing

This study reveals that the prevalence of PPD in women within 1 year postpartum in the urban areas of Beijing is 29.42%. Compared to the global prevalence of PPD, which ranges from 2.1 to 31.6% (22, 23), and recent surveys in China indicating a detection rate of PPD between 3.7 and 34.7%, this figure is notably high (6). The variance in reported PPD prevalence rates can be attributed to the use of different assessment tools and cut-off values (6, 22, 23), highlighting the need for standardized methods in future research. Meanwhile, this study included postpartum women living and working in the six urban districts of Beijing. The high incidence of PPD in urban Beijing may be attributed to the fast pace of life, intense competition, and significant work and economic pressures (24).

### 4.2 Occupational differences and PPD prevalence

In this study, the prevalence of PPD among women employed in commercial enterprises was found to be 39.81%, significantly higher than that of physical workers (18.92%), professionals, government office & public institution employees (21.97%), and the unemployed or self-employed (28.71%). This disparity underscores the substantial differences in the prevalence of PPD among postpartum women of various occupations, highlighting the necessity of focusing on PPD from an occupational perspective. Women working in commercial enterprises are often exposed to greater pressures of competition and unemployment, which might be associated with higher rates of PPD (25, 26), and their psychological state, particularly postpartum mental health, requires special attention. In this study, women in commercial enterprises tended to be older compared to other groups, which may be associated with the higher incidence of PPD. Research has found that older postpartum women have a higher incidence of post-pregnancy complications and poorer postpartum recovery; on the other hand, older mothers tend to have more concerns, which may lead to greater mental stress, resulting in anxiety, depression, and other adverse emotional states (27, 28). Furthermore, the proportion of first-time mothers was higher among those in commercial enterprises. As novice mothers, the lack of experience in childbirth and postpartum baby care can also lead to postpartum depression (29, 30).

### 4.3 Multiple factors influencing PPD among working women

Further multivariate analysis revealed that women working in commercial enterprises are affected by a wider range of factors contributing to PPD. Among these, personal monthly income of 5,000–15,000 RMB was identified as risk factors. Multiple studies have found that PPD is related to the family’s economic status (5, 6, 27, 31, 32). Both higher and lower family incomes may increase the incidence of PPD. This is consistent with the findings in this study regarding women in physical labor occupations, where family monthly income affects PPD. In this study, although the family monthly income of women in commercial enterprises was significantly higher than that of other occupational categories, no correlation was found with the occurrence of PPD. In contrast, the personal monthly income of the women was related to PPD. This suggests that women in commercial enterprises may focus more on their career development, which could lead to increased stress.

This study also found that maternity leave of less than 6 months significantly increases the risk of PPD for women in commercial enterprises. This aligns with the findings of other studies (9, 10), which indicate that shorter maternity leave is associated with higher rates of postpartum depression. Adequate maternity leave is associated with improved recovery for the mother and potentially better care of the newborn. Therefore, ensuring sufficient maternity leave is an important way to protect the postpartum physical and mental health of working women (33). In China, maternity leave for mothers is divided into three parts: basic maternity leave, extended maternity leave, and local maternity leave. According to China’s labor law regulations, female employees are entitled to 98 days of paid maternity leave. In special circumstances, such as in cases of dystocia, the leave can be extended by an additional 15 days. And in Beijing, female employees also enjoy an additional local 60 days of maternity leave. However, in this study, maternity leaves shorter than 6 months were positively correlated with postpartum depression in women in commercial enterprises, indicating that current maternity leave policies require reasonable adjustments based on the specific characteristics of the occupation, and from the perspective of postpartum mothers’ mental health.

There is an association between the method of infant feeding and the psychological state of the mother. In this study, compared to exclusive breastfeeding, mixed or formula feeding resulted in a higher prevalence of PPD among women in commercial enterprises and in professionals, government office & public institution employees. Conversely, for unemployed and self-employed women, mixed or formula feeding resulted in a lower prevalence of PPD. This may be because mixed or formula feeding, being more complex and troublesome, can increase anxiety more easily in those working women facing greater occupational stress (7). In Chinese culture, breastfeeding often extends up to 1 year or more. For working women, this practice poses significant challenges. Once maternity leave concludes, lactating women are required to frequently travel between home and workplace to breastfeed, a process that is both time-consuming and physically draining, thereby exerting considerable psychological pressure (34, 35). Alternatively, many postpartum mothers returning to work opt to express breast milk using pumps and temporarily store it at the workplace for later use. However, societal stigmas surrounding breastfeeding, coupled with unfriendly and inadequate facilities at work, exacerbate the mental stress experienced by professional women (36). This situation underscores the need for more supportive breastfeeding environments tailored to the needs of working women (37, 38).

The study also found that having relatives help with care is a protective factor against PPD. The presence of relatives to assist with care is associated with a lower prevalence of PPD among women in commercial enterprises and those in professionals, government office & public institution employees. The level of postpartum support is considered one of the most important influencing factors for PPD (39–41). This is particularly crucial for mothers returning to the workforce. Compared to hiring nannies, care and attention from family members provide the best psychological support for postpartum women. For Chinese parents, options for infant care are limited. Before the age of three, due to the lack of affordable public infant and toddler care facilities, caregiving predominantly takes place at home. Hiring a nanny entails additional financial burdens and concerns about the caregiver’s qualifications. On the other hand, entrusting grandparents with childcare often leads to increased family conflicts. This scenario underscores the need for government bodies and workplaces to provide high-quality, trustworthy childcare services, which are particularly crucial for postpartum working women. The establishment of such services would alleviate the caregiving burden on families and support the mental and emotional well-being of working mothers.

### 4.4 Limitations

While this study provides valuable insights into the prevalence and factors associated with PPD in different occupational groups, it has its limitations. As a cross-sectional study, it cannot establish causality between identified risk factors and PPD. Future longitudinal studies are recommended to address this gap. Moreover, a more comprehensive analysis of occupational stress and its impact on PPD would enhance our understanding of the occupational-specific risks faced by these women. The reliance on self-reported data may introduce recall bias, suggesting the need for more objective measurement tools in future studies.

## 5 Conclusion

In conclusion, the higher prevalence of PPD in women employed in commercial enterprises, influenced by various occupational and personal factors, calls for further research and targeted intervention strategies. This study, with its focus on occupational factors, provides a novel perspective in analyzing the risk factors for PPD among working women in Beijing. The findings offer theoretical support for developing effective screening and intervention measures tailored to the needs of women in different occupations, aiming to promote their mental health and well-being postpartum.

## Data availability statement

The original contributions presented in the study are included in the article/supplementary material, further inquiries can be directed to the corresponding author.

## Author contributions

JZ: Data curation, Investigation, Methodology, Software, Writing – original draft. MZ: Conceptualization, Supervision, Writing – review & editing.

## Ethics statement

The studies involving humans were approved by the Ethics Review Committee of the Institute of Basic Medical Sciences, Chinese Academy of Medical Sciences. The studies were conducted in accordance with the local legislation and institutional requirements. The participants provided their written informed consent to participate in this study. Written informed consent was obtained from the individual(s) for the publication of any potentially identifiable images or data included in this article.
